# Side-by-side regions in dorsolateral prefrontal cortex estimated within the individual respond differentially to domain-specific and domain-flexible processes

**DOI:** 10.1152/jn.00277.2023

**Published:** 2023-11-08

**Authors:** Lauren M. DiNicola, Wendy Sun, Randy L. Buckner

**Affiliations:** ^1^Department of Psychology, Center for Brain Science, Harvard University, Cambridge, Massachusetts, United States; ^2^Division of Medical Sciences, Harvard Medical School, Boston, Massachusetts, United States; ^3^Athinoula A. Martinos Center for Biomedical Imaging, Massachusetts General Hospital, Charlestown, Massachusetts, United States; ^4^Department of Psychiatry, Massachusetts General Hospital, Charlestown, Massachusetts, United States

**Keywords:** association cortex, BA9/46, cognitive control, frontoparietal control network

## Abstract

A recurring debate concerns whether regions of primate prefrontal cortex (PFC) support domain-flexible or domain-specific processes. Here we tested the hypothesis with functional MRI (fMRI) that side-by-side PFC regions, within distinct parallel association networks, differentially support domain-flexible and domain-specialized processing. Individuals (*N* = 9) were intensively sampled, and all effects were estimated within their own idiosyncratic anatomy. Within each individual, we identified PFC regions linked to distinct networks, including a dorsolateral PFC (DLPFC) region coupled to the medial temporal lobe (MTL) and an extended region associated with the canonical multiple-demand network. We further identified an inferior PFC region coupled to the language network. Exploration in separate task data, collected within the same individuals, revealed a robust functional triple dissociation. The DLPFC region linked to the MTL was recruited during remembering and imagining the future, distinct from juxtaposed regions that were modulated in a domain-flexible manner during working memory. The inferior PFC region linked to the language network was recruited during sentence processing. Detailed analysis of the trial-level responses further revealed that the DLPFC region linked to the MTL specifically tracked processes associated with scene construction. These results suggest that the DLPFC possesses a domain-specialized region that is small and easily confused with nearby (larger) regions associated with cognitive control. The newly described region is domain specialized for functions traditionally associated with the MTL. We discuss the implications of these findings in relation to convergent anatomical analysis in the monkey.

**NEW & NOTEWORTHY** Competing hypotheses link regions of prefrontal cortex (PFC) to domain-flexible or domain-specific processes. Here, using a precision neuroimaging approach, we identify a domain-specialized region in dorsolateral PFC, coupled to the medial temporal lobe and recruited for scene construction. This region is juxtaposed to, but distinct from, broader PFC regions recruited flexibly for cognitive control. Region distinctions align with broader network differences, suggesting that PFC regions gain dissociable processing properties via segregated anatomical projections.

## Graphical abstract

**Figure:**
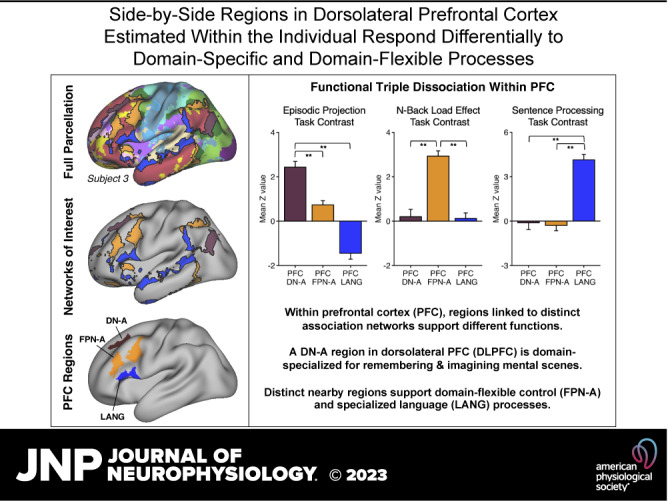

## INTRODUCTION

Prefrontal cortex (PFC) is a heterogeneous structure that, among other functions, supports maintaining information and goals in mind and flexibly adapting behavior to changing environmental demands (1–11). PFC develops slowly after birth (e.g., Ref. 12) and has expanded in primate evolution, with apes and humans possessing a particularly large and complex PFC (13, 14). Analysis of cytoarchitectonic features reveals multiple cortical subregions in PFC (e.g., Refs. 15, 16) that also vary in connectivity (e.g., Refs. 16–18), raising questions about both differentiation of PFC and the role that specialized subregions play in later-developing, higher-order functions.

A prominent debate about PFC function contrasts domain-flexible with domain-specific processing. Early anatomical studies in the rhesus monkey noted that rostral regions of PFC are positioned as convergence zones receiving and integrating projections from multiple sensory systems (19, 20). One influential model posits that lateral PFC exerts domain-flexible control over domain-specialized, posterior cortical regions via biasing signals (4, 21). This model is supported by neurophysiological work showing multidomain neuronal responses during working memory tasks that adapt flexibly to current task demands (e.g., Refs. 22, 23). Within this model, convergence is emphasized, with PFC as an apex control zone that adjusts its representational properties in a domain-flexible manner (4). Human neuroimaging findings, especially description of the domain-flexible (multiple demand) network that prominently includes lateral PFC, are consistent with this hypothesis (24, 25).

An alternative hypothesis emphasizes that domain specificity is maintained within PFC, including regions that represent spatial information. Drawing upon neuroanatomical work showing distinct connectivity of side-by-side PFC regions with multiple (sometimes juxtaposed) regions of temporal and posterior parietal cortex, Goldman-Rakic and colleagues (26, 27) advanced a model that proposes that distinct regions of PFC are specialized for processing domains. This model is supported by differential domain selectivity of sustained neuronal responses in distinct regions of PFC during delayed-response tasks (e.g., Refs. 27, 28). Within this alternative model, regions of PFC gain and maintain information selectivity via participation in distinct, parallel distributed networks.

Insight into these divergent views of PFC function has emerged from the study of human language. Lesions to inferior PFC that are in the vicinity of the historically defined “Broca’s area” lead to production impairments (see Refs. 29, 30). Neuroimaging work supports a role of inferior PFC in language-relevant tasks (i.e., making associations based on word meaning; e.g., Refs. 31, 32). However, paralleling the debate between domain-flexible and domain-specific functions of PFC in monkeys, other neuroimaging studies find that regions at or near Broca’s area respond when control demands are increased, even for tasks that do not require processing of word meaning, raising the possibility that domain-flexible control demands embedded in commonly used language tasks drive inferior PFC response (Ref. 33; see also Refs. 34–36).

Fedorenko and colleagues (37) provided a result that helps reconcile these divergent findings: when studied within the detailed anatomy of the individual, domain-flexible control regions were discovered to be right next to, and easy to blur with, distinct regions responsive selectively to meaning-based sentence processing. These results from within-individual precision neuroimaging raise the possibility that domain-flexible and domain-specialized regions of inferior PFC may both exist and contribute distinct functions (38).

In the present study, we adopt within-individual precision neuroimaging approaches to revisit functional specialization of human lateral PFC. Our study anchors from the possibility that a domain-specific region of dorsolateral PFC (DLPFC) may be juxtaposed next to a larger domain-flexible region of DLPFC that extends across much of lateral PFC. This possibility is raised by the small DLPFC region consistently observed in analyses of networks coupled to posterior parahippocampal cortex (39), distinct from regions associated with cognitive control (40).

We first set out to identify candidate regions across PFC, defined by their functional connectivity to distinct association networks (e.g., Refs. 41, 42; see also Refs. 43, 44). We then tested the functional properties of the side-by-side regions of PFC, using task contrasts designed to distinguish domain-flexible cognitive control from domain-specialized processing functions. As the results reveal, we replicate the double dissociation noted by Fedorenko and colleagues (37) in inferior PFC. We further discover that a specific region of DLPFC, coupled to the medial temporal lobe (MTL), is active during remembering and imagining the future, preferentially for processing scene/spatial information. This region is distinct from (and easily confused with) a large, juxtaposed region of lateral PFC that responds to domain-flexible demands on cognitive control.

## MATERIALS AND METHODS

### Participants

Nine paid participants ages 19–27 yr were recruited from the Boston area [mean age = 21.4 yr (SD = 2.8), 8 right-handed, 4 identifying as female] and completed a behavioral session (to practice tasks) and at least two neuroimaging sessions. All participants provided written informed consent, and all study protocols were approved by the Harvard University Institutional Review Board.

### MRI Data Acquisition and Processing

Neuroimaging data in this article are new and presented for the first time. Neuroimaging took place at the Harvard Center for Brain Science with a 3-T Siemens Magnetom Prisma-fit MRI scanner and 32-channel head coil (Siemens Healthcare, Erlangen, Germany). A T1-weighted (T1w) structural image (1.2-mm voxels, TR = 2,200 ms, TE = 1.57, 3.39, 5.21, 7.03 ms, TI = 1,100 ms, 144 slices, flip angle = 7°, matrix = 192 × 192, in-plane GRAPPA acceleration = 4) was acquired with a multiecho magnetization-prepared rapid acquisition gradient-echo sequence [ME-MPRAGE; van der Kouwe et al. (83)]. Blood oxygenation level-dependent (BOLD) data were acquired with a multiband gradient-echo echo-planar pulse sequence (e.g., Refs. 45, 46; provided by the Center for Magnetic Resonance Research at the University of Minnesota; 2.4-mm voxels, TR = 1,000 ms, TE = 33 ms, flip angle = 64°, matrix = 92 × 92, 65 slices). During BOLD runs, participants’ eyes were video monitored for assignment of a “sleepiness score” with the EyeLink 1000 Core Plus with Long-Range Mount (SR Research, Ottawa, ON, Canada).

Data were processed with a custom pipeline (“iProc”; see Ref. 47 for details), integrating tools from FreeSurfer (v6.0; Ref. 48), FSL (v5.0.4; Ref. 49), and AFNI (50). Data were aligned to each participant’s T1w structural image through a single interpolation, with the goal of minimizing blurring and preserving idiosyncratic anatomical details. The T1w target was verified to have a good pial and white matter boundary estimation (output by FreeSurfer recon-all). The single interpolation combined four matrices: motion correction [to a run’s middle volume, with FSL MCFLIRT and 12 degrees of freedom (DOF)], field map unwarping (with FSL FUGUE), registration to a mean BOLD template (with FSL FLIRT and 12 DOF), and registration of the mean BOLD to a T1w image (with FreeSurfer boundary-based registration and 6 DOF). To create a mean BOLD template, field map-unwarped middle volumes of all runs were first registered to a temporary target, the unwarped middle volume of a single run (upsampled to 1.2 mm), and then averaged.

After alignment, nuisance variables (6 motion parameters, whole brain, ventricular, and white matter signals and their temporal derivatives) were regressed from the data to be used for functional connectivity network estimation, which were then band-pass filtered (0.01–0.10 Hz, with AFNI). Whole brain signal was also regressed from BOLD data for the task contrasts (as in Ref. 43). All BOLD data were resampled to the fsaverage6 cortical surface mesh and smoothed with a 2-mm full-width at half-maximum kernel (with FreeSurfer vol2surf and surf2surf).

### “Resting-State” Fixation Data

Data during eyes-open fixation were acquired to be used for network estimation. Each participant completed six runs, three per session (7 min 2 s each; 42 min 12 s total), during which they fixated on a black crosshair at the center of their view. Fixation runs occurred nonconsecutively in each session.

### Task Paradigms

Tasks were selected from the existing literature based on their ability to dissociate domain-flexible from domain-specific processing functions and to do so within individuals through repeated scanning (i.e., multiple runs of each task) to boost the signal-to-noise ratio (SNR). Specifically, we targeted multiple hypothesized dissociations. The first contrasted language-specific processing and domain-flexible control (replicating Refs. 37, 51). The second, novel dissociation contrasted remembering/spatial processes and domain-flexible control (building from Refs. 43, 52). Domain-flexible cognitive control was targeted with a working memory (N-Back) task adapted from the Human Connectome Project (HCP; Ref. 53).

Tasks were presented with PsychoPy (v2021.1.4). Participants viewed task stimuli through a mirror attached to the scanner head coil. The stimulus viewing location was adjusted to ensure a comfortable viewing angle. Before each task run, participants were reminded to stay still and alert. All stimuli were displayed as black on a light gray background except for during the N-Back task, when images or white text were shown on a black background (matching presentation in the HCP). All task runs began with 12 s of fixation for T1 stabilization. See Supplemental Materials for example stimuli.

#### Working memory (N-Back) task.

Demands on cognitive control were manipulated in a working memory (N-Back) task that varied memory load from a low-load condition (0-Back) to a high-load condition (2-Back). In our targeted analysis, the primary contrast used Letter stimuli. Stimulus categories were manipulated across blocks to vary information content (i.e., Letters, Words, Faces, Scenes), enabling a secondary analysis to test whether effects generalized across categories.

Participants completed eight runs of the N-Back task, four per session (4 min 44 s each; 37 min 52 s total). Our task was modeled after the HCP (see Ref. 53), including timing, load level, and use of multiple stimulus categories. During each run, participants viewed stimuli across all four categories and indicated whether each stimulus matched either an initial cue (0-Back condition) or the stimulus seen two prior (2-Back condition). The block type (0-Back or 2-Back) was cued with the first stimulus in each block. Runs began with 12 s of fixation, followed by eight extended N-Back trial blocks (one 0-Back and one 2-Back per category per run). Each trial block lasted 25 s, including the 2-s cue and nine 2.5-s trials (2.0-s stimulus presentation followed by 0.5-s intertrial interval). Fixation blocks (15 s each) also appeared after every other trial block.

Within each N-Back trial block, two stimuli were targets and two were nontarget lures. Targets in the Face, Scene, and Letter categories were identity matches; targets in the Word category were rhyming words. Lures in the 2-Back condition were repeats or rhymes in the wrong position; lures in the 0-Back condition were repeats or rhymes that did not match the cue. 0-Back and 2-Back condition and stimulus category were each counterbalanced across blocks; no categories appeared twice in a row. Target and lure positions were counterbalanced across and within runs. Participants responded “Match” (right index finger) or “No-Match” (left index finger) for each trial. Face stimuli were those provided by the HCP (53). Scene stimuli were from the Konkle lab (54, 55). Letters were consonants. Words were one-syllable words, organized into 10-word sets matched for length and frequency using the Corpus of Contemporary American English (Ref. 56, December 2015 version).

The N-Back load effect (2-Back vs. 0-Back) for the Letter category was our primary target for analysis, which we hypothesized would preferentially recruit PFC regions of a frontoparietal network (termed FPN-A^1^). This contrast was chosen a priori. In follow-up analyses, we also examined the load effect across each of the four categories, to further explore domain flexibility.

#### Sentence Processing task.

The Sentence Processing task was developed by Fedorenko et al. (37, 51) to examine domain-specific processing related to accessing word meaning and phrase-level meaning. The target task involved real word strings that formed sentences presented one word at a time. The reference control task was presentation of nonword strings that were matched in length and were visually similar.

Each session included two runs of the Sentence Processing task, for a total of four runs (5 min 0 s each; 20 min 0 s total). During each task block, participants passively read word strings forming sentences or sequences of pronounceable nonwords (Nonwords). Each run included 12 blocks of word strings (6 Sentence and 6 Nonword). Blocks lasted 18 s and featured three strings, each with 12 (non)words onscreen for 0.45 s. At the end of each string, participants saw a cue (0.5 s) and pressed a button to indicate that the word string was complete (right index finger). The Sentence and Nonword conditions were equally likely to appear in every order position and did not appear more than twice in a row. Runs also included four fixation blocks (18 s), at the beginning of each run and after every fourth task block.

The Sentence versus Nonword effect was our primary target for analysis of domain-specific regions relevant to language and has been shown to preferentially recruit a language network (termed LANG) in recent work (Ref. 57; see also Refs. 37, 51). We specifically hypothesized that the Sentence versus Nonword contrast would recruit PFC regions of the LANG network.

#### Episodic Projection task.

The Episodic Projection task was designed to target processing linked to remembering and imagining the future (prospection) through a contrast that has selectively activated default network A (DN-A) in prior studies (averaging contrasts of Past Self vs. Present Self and Future Self vs. Present Self as in Ref. 43). The task requires participants to view and respond to questions about brief scenarios referring, for example, to possible past and future events or current feelings (see also Ref. 84). This is a complex task contrast with multiple component processes. DiNicola et al. (52) further demonstrated that DN-A responds to the extent to which individual trials encourage participants to mentally construct spatial scenes (termed Scene Construction; Ref. 59). In our targeted analysis, the primary contrast used condition-level (block) analysis (mimicking the Episodic Projection conditions in Table 1 of Ref. 43). Secondary analyses explored the degree to which trial-level responses varied in relation to processes associated with scene construction and trial difficulty (using all 180 trials from the extended set of Episodic Projection conditions as in Table 3 of Ref. 43).

Participants completed six runs of the Episodic Projection task, three per session (10 min 17 s each; 61 min 42 s total). During each run, participants read 30 brief scenarios and then viewed a question. Trials varied in self-relevance (Self vs. Non-Self) and time frame (Past, Present, or Future). Participants were instructed to consider each of three potential responses and to select the best choice (left hand keypress). Five questions from each of three relevant conditions (Past Self, Present Self, and Future Self) were included per run [10-s trial, 10-s interstimulus interval (ISI)], in a randomized order that remained consistent across participants. Additional trial conditions, with equal numbers of intermixed trials, included trials from the Past Non-Self, Present Non-Self, and Future Non-Self trials of DiNicola et al. (43). These later trial types were not used in the primary condition-level (block) analyses but were used in secondary trial-level analyses. Questions from a single condition did not appear more than twice in a row.

The Past Self versus Present Self and Future Self versus Present Self contrasts were averaged, and this contrast was the primary target for analysis. We hypothesized that PFC regions of the DN-A network would be preferentially recruited. Follow-up analyses examined trial-level effects to further probe the domain specificity of the response.

### Data Inclusion and Quality Control

Data were examined and excluded for quality. All exclusions were made before examination of the functional task responses. Exclusion criteria included maximum absolute motion > 1.8 mm (cumulatively across a run) and slice-based signal-to-noise ratio < 135. Across the nine analyzed participants (*S1–S9*), the data quality was high, with a total of four runs (out of 216) excluded because of motion across participants. *S1* had three runs (1 each of Sentence Processing, N-Back, and Fixation) excluded and *S7* had one Episodic Projection run excluded because of motion. For *S9*, a scanner error (i.e., that temporarily changed the bounding box of the acquired data) led to the exclusion of one Sentence Processing run. Two runs with maximum motion of 2.0 mm (deemed gradual) were included for S9 (one Episodic Projection and one Fixation). One run was inadvertently excluded from the Episodic Projection task contrast analysis for both S6 and S7 (with no detectable effect on results).

Behavioral performance was also examined. Runs with “sleepiness scores” higher than 1 (alert throughout) were investigated, as were runs with skipped trials. For *S2*, one N-Back run was excluded because of consecutive skipped trials. No other runs were excluded based on behavioral performance. Within the Episodic Projection task, for two participants (*S6* and *S7*) a question was erroneously repeated within a run. This trial was excluded from both condition- and trial-level analyses. For Episodic Projection trial-level analysis, to be conservative, trials without recorded responses were excluded even if participants were alert throughout the run. Overall, *S2* had two trials, *S5* had one trial, and *S6* had two trials (each out of 180) excluded because of nonresponses.

### Network Identification

Network estimation was completed with a multisession hierarchical Bayesian model (MS-HBM; see Ref. 60) as implemented in Du et al. (61). For network estimation, data were modeled in independent groups of five and six participants, which yielded a within-individual parcellation for each participant.^2^ Two participants included in the network groupings did not collect usable Sentence Processing data and therefore were not included in the task-based analyses.

Briefly, for each individual, the MS-HBM assigned all vertices on the fsaverage6 surface to 1 of 15 networks as developed and validated by Du et al. (61). Each individual’s available fixation runs were input to the model. First, correlations between each surface vertex (40,962 in total) to 1,175 regions of interest (distributed across an fsaverage surface) were defined (see Ref. 62). The top 10% of correlations were preserved as an initial functional connectivity profile (60, 62). The MS-HBM was initialized with, for each group, the profiles of the relevant individuals and a 15-network group-level prior (generated with data from the HCP S900 data release as reported in Ref. 61). Since our goal was network estimation, rather than training and validation, we did not run a validation step (described in Ref. 60). Parcellations from the MS-HBM “training” step were carried forward.

The present analysis targeted three networks: Default Network-A (DN-A), Frontoparietal Network-A (FPN-A) and a Language Network (LANG). DN-A was of particular interest because of its relation to the prior work of Vincent et al. (39, 40) that suggested a DLPFC region coupled to the parahippocampal cortex (e.g., see Fig. 8 in Ref. 39). DN-A is the more refined network estimate that emerges in precision within-individual estimates of the network (e.g., Ref. 42). In all individuals, the model estimates of these networks included expected referential features (e.g., Refs. 42, 43, 47, 57). DN-A, for example, included the posterior parahippocampal cortex as well as posterior parietal cortex and posteromedial cortex, where a triad of regions often surrounds a region of another network (DN-B). LANG included inferior frontal gyrus, superior temporal cortex, and posterior superior frontal gyrus (see Refs. 51, 57). FPN-A included multiple PFC regions extending across DLPFC and toward/along the inferior frontal gyrus, as well as regions at or near the intraparietal sulcus and in the anterior cingulate (e.g., Refs. 42, 57, 58). The model networks were checked by examining seed region correlations (e.g., see Ref. 42).

### Construction of Hypothesis-Targeted Prefrontal Regions

**Figure 1.**
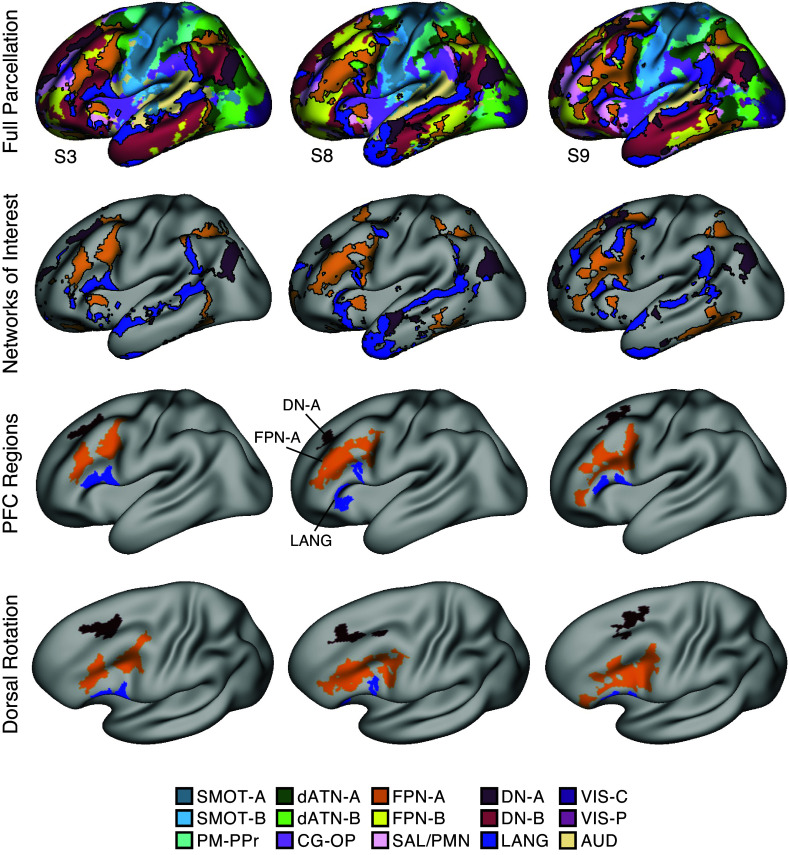
Three side-by-side lateral prefrontal regions were identified within each individual based on functional connectivity to distinct networks. Each column displays an example within-individual estimate of networks and derived lateral prefrontal cortex (PFC) regions. *Top*: full cerebral network estimates generated by a multisession hierarchical Bayesian model (MS-HBM) are shown. The key at *bottom* displays the color labels and abbreviated names for the 15 estimated networks based on Du et al. (61). *Second row*: although the exact anatomical positioning varied, the 3 networks of interest, Default Network-A (DN-A, maroon), Frontoparietal Network-A (FPN-A, orange), and a language network (LANG, blue), could be identified across all tested participants. *Third row*: within each network, 3 lateral PFC regions were identified and isolated in both hemispheres. These regions, which were defined a priori before processing the task data, were used for subsequent analyses. *Bottom*: surface images are tilted for a better view of dorsolateral PFC (DLPFC). This view is also used in Fig. 5. Images show the left hemisphere, but the regions were bilaterally defined. Network estimates and PFC regions for all individuals, in both hemispheres, can be found in the Supplemental Materials. Network labels match those in Ref. 61: SMOT-A, Somatomotor-A; SMOT-B, Somatomotor-B; PM-PPr, Premotor-Posterior Parietal Rostral; CG-OP, Cingular-Opercular; SAL / PMN, Salience / Parietal Memory Network; dATN-A, Dorsal Attention-A; dATN-B, Dorsal Attention-B; FPN-A, Frontoparietal Network-A1; FPN-B, Frontoparietal Network-B; DN-A, Default Network-A; DN-B, Default Network-B; LANG, Language; VIS-C, Visual Central; VIS-P, Visual Peripheral; AUD, Auditory. Data were visualized, and prefrontal region masks created, using Connectome Workbench v1.3.2 (85).

For each individual, after DN-A, FPN-A, and LANG networks were identified, regions of each network within lateral PFC were defined bilaterally: a DLPFC region in DN-A, a region in FPN-A extending from DLPFC to the inferior frontal gyrus, and an inferior PFC region in LANG. Given individual differences in network topography, unique regions were defined within each individual separately. Boundary vertices (i.e., directly between PFC regions for the 3 networks) were excluded to account for some level of unavoidable spatial blurring. The region associated with FPN-A included the greatest number of vertices, but PFC regions in all three networks were successfully identified, in both hemispheres, in all participants (see also Supplemental Materials). Figure 1 illustrates example PFC regions. Note that the region is bilateral but illustrated only for the left hemisphere. Although we analyzed the data from the bilateral regions, post hoc analyses revealed that the effects were also present when analyses were restricted to each hemisphere (see Supplemental Materials).

### Task Analysis

#### Testing the hypothesized triple dissociation.

We hypothesized a triple functional dissociation among lateral PFC regions, with the DLPFC regions linked to DN-A preferentially recruited by the Episodic Projection task contrast, FPN-A by the N-Back Load Effect task contrast, and LANG by the Sentence Processing task contrast. The critical hypothesis was tested first by exploring the full 3 × 3 interaction via a repeated-measures ANOVA and then through post hoc tests to show that each direct contrast supported the triple dissociation (using R *stats* v4.1.1). Given the a priori hypotheses, the post hoc tests were one tailed.

To create the contrasts, surface-projected task data were analyzed within individuals in hemisphere-specific, run-level general linear models (GLMs; created with FSL first-level Feat). A canonical (double gamma) hemodynamic response function and its temporal derivative as well as a high-pass filter (100 s/0.01 Hz cutoff) were used in all models.

For the Episodic Projection task, the GLM was constructed with conditions as regressors (producing *z-*statistical maps for condition contrasts). Results from run-specific, condition-level GLMs included maps for the two contrasts of interest, Past Self versus Present Self and Future Self versus Present Self, which were averaged to produce a mean *z*-statistical “Episodic Projection” contrast map. For the N-Back task, the GLM featured regressors for each trial block (including the cue stimulus). Using only the Letter category, *z*-statistical maps for relevant blocks were averaged within a load level (2-Back or 0-Back). Taking the difference (2-Back minus 0-Back) produced a mean *z*-statistical “N-Back Load Effect” contrast map. For the Sentence Processing task, the GLM included conditions as regressors and produced *z*-statistical maps for the Sentence versus Nonword contrast of interest. These maps were averaged across runs to produce a mean *z*-statistical “Sentence Processing” contrast map.

For each individual, mean *z* values were extracted from each of these three contrast maps from the a priori-defined PFC regions using masks. This allowed for calculation of three mean *z* values per task contrast, representing activity within the separate PFC regions linked to the DN-A, FPN-A, and LANG networks.

#### Trial-level analyses to dissociate Scene Construction from Difficulty.

For the Episodic Projection task, trial-level variation provided an additional opportunity to probe functional dissociation between PFC regions linked to DN-A and FPN-A, the novel dissociation unpacked in this paper. Specifically, each trial of the Episodic Projection task is associated with its own level of Difficulty and also a level of reliance on Scene Construction (i.e., imagining the locations of objects and places). These trial-level processing demands have previously been measured by DiNicola et al. (52). Here, we extracted mean *z* values for each trial from each of the PFC regions by coding trials separately within the GLM. These *z* values were then averaged across participants, so each PFC region had a single mean value for each trial. We used all 180 trials available, paralleling DiNicola et al. (52) and maximizing the dispersion of the trial-level values. Each trial included data from at least 7 participants (with all but three trials including data from at least 8 and over 80% of trials from all 9 participants). We hypothesized that the regions of PFC associated with DN-A would positively correlate with Scene Construction ratings and the regions associated with FPN-A with Difficulty ratings (using ratings from Exp. 2 in Ref. 52). We also tested the extent to which behavioral composite scores predicted activity, using region-specific multiple regression models, as in our prior work (i.e., featuring composites for Scene Construction, Difficulty, and Others-Relevant strategies^3^; Ref. 52).

#### N-Back Load Effect analysis across categories.

The analyses proposed to this point seek evidence for functional dissociation between regions, leveraging differential responses to distinct processing domains. PFC regions linked to cognitive control are commonly hypothesized to increase their response to effort/difficulty across multiple information processing domains (7, 9, 24). To probe the domain flexibility of the PFC region linked to FPN-A, in post hoc analyses we analyzed the Load Effect separately for the Scene, Face, and Word conditions of the N-Back task, in addition to the Letter condition utilized in the earlier analyses. We aimed to characterize the degree to which the PFC region linked to FPN-A would respond to the Load Effect generalizing across domains and do so in a way that consistently differentiated response properties from the regions linked to DN-A and LANG.

#### Contrast visualization.

The analyses above define a priori PFC regions within networks. To further explore the spatial patterns of the task contrasts without spatial assumptions, patterns of activity across three critical task contrasts were plotted in relation to the full boundaries of the networks of interest. For these analyses, we focus on task contrasts that isolate the domain-preferential effects separate from the domain-flexible N-Back Load Effect.

Specifically, we created a Scene Construction contrast, using the 30 trials with the highest and the 30 trials with the lowest Scene Construction scores (using ratings from Exp. 2 of Ref. 52). Trial-level *z*-statistical maps were averaged within each set, and a difference map (“high” minus “low”) produced the Scene Construction contrast map. The N-Back Load Effect contrast map represented the overall load effect, created using 2-Back versus 0-Back blocks from all four categories (Letter, Face, Scene, and Word). The Sentence Processing contrast maps contrasted the “Sentence” strings to the “Nonword” strings (identical to the primary analysis above).

Within individuals, the Scene Construction, N-Back Load Effect, and Sentence Processing contrast maps were visualized in relation to the boundaries of DN-A, FPN-A, and LANG. Within these maps, the threshold range preserved high-confidence activations (similar to Ref. 57).

## RESULTS

### Juxtaposed Regions in Prefrontal Cortex Show Robust Functional Dissociation

Our central hypothesis is that distinct, side-by-side regions of PFC participate differentially in domain-specific and domain-flexible processing functions, including within the broad region of DLPFC. Analysis of a priori-defined PFC regions associated with DN-A, FPN-A, and LANG revealed the hypothesized triple dissociation, with the DN-A DLPFC region preferentially recruited by the Episodic Projection task contrast, the FPN-A PFC region by the N-Back Load Effect contrast, and the LANG PFC region by the Sentence Processing contrast. This robust effect was supported by statistical tests.

**Figure 2.**
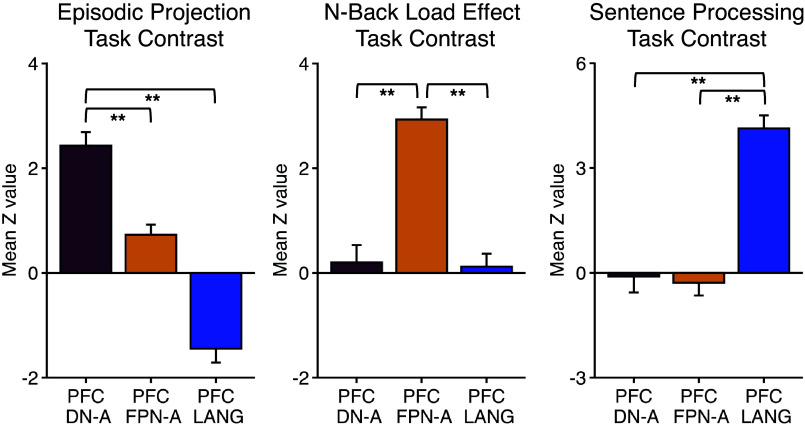
Functional task responses reveal a robust triple dissociation among juxtaposed lateral prefrontal regions. Each panel displays the mean response for a single task contrast for each of the 3 anatomically distinct regions of prefrontal cortex (PFC). *Left*: the Episodic Projection task contrast shows a robust response for the PFC region linked to Default Network-A (DN-A) that is greater than the other 2 regions. *Center*: the N-Back Load Effect task contrast shows a large and differential response in the PFC region linked to Frontoparietal Network-A (FPN-A). *Right*: the Sentence Processing task contrast preferentially recruits the PFC region linked to the language network (LANG). Mean activity values were first taken within each individual’s specific PFC region; the mean and SE of values across individuals were then plotted. Within each contrast, paired *t* tests between regions support the observed differences (**P < 0.001). The full interaction was significant (*P* < 0.001).

A repeated-measures ANOVA on region-level activity revealed a significant interaction between the effects of task contrast and PFC region [*F*(4,32) = 63.06, *P* < 0.001; see Fig. 2]. Paired *t* tests then quantified, for each contrast, comparisons between pairs of PFC regions. The Episodic Projection contrast preferentially recruited the DN-A region over those of FPN-A [*t*(8) = 10.56, *P* < 0.001] and LANG [*t*(8) = 11.02, *P* < 0.001]. The N-Back Load Effect contrast (Letters category) recruited the FPN-A region over those of DN-A [*t*(8) = 5.78, *P* < 0.001] and LANG [*t*(8) = 7.28, *P* < 0.001], and the Sentence Processing contrast recruited the LANG PFC region over those of DN-A [*t*(8) = 7.73, *P* < 0.001] and FPN-A [*t*(8) = 7.55, *P* < 0.001], confirming the triple dissociation.^4^

These PFC regional response patterns matched those observed for the broader networks (see Supplemental Materials), supporting that the functional response properties of the PFC regions align with the response properties of the distributed networks to which they belong. The PFC regional response patterns were also present in hemisphere-specific analyses and in analyses using group-level PFC regions created using the model prior (see Supplemental Materials). Thus, although we employed a precision approach to maximize the anatomical details within individuals, and bilateral regions were selected for tests a priori, the effects are robust and generalize across all tested variations of alternative post hoc analyses.

### A Specific Region of Dorsolateral Prefrontal Cortex Responds to Scene Construction

We analyzed responses to trial-level processing demands to further explore the functional dissociation between the DLPFC region associated with DN-A, found to be preferentially activated by the Episodic Projection task contrast, and the adjacent PFC region linked to FPN-A. Specifically, we utilized estimates of each trial’s reliance on Scene Construction as well as each trial’s level of Difficulty. Here we used all available trials (*N* = 180), similar to DiNicola et al. (52), to increase the dispersion of the trial-level estimates. The MRI data here are new and independent of the data explored in the earlier 2023 report. We again found clear support for a double dissociation, with additional evidence for the specific role of the DLPFC region of DN-A in scene construction (59).

**Figure 3.**
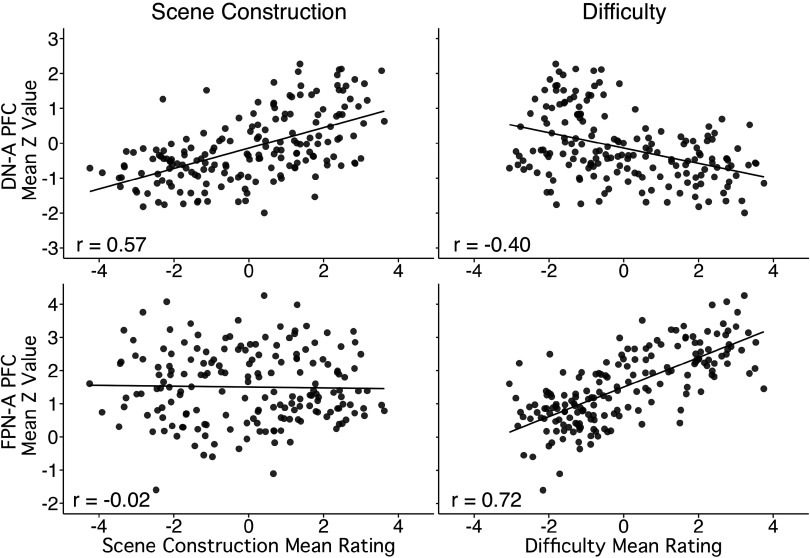
Trial-level analyses reveal a dorsolateral prefrontal region that selectively increases response to Scene Construction but not Difficulty. Each scatterplot displays the relation between component process levels and regional responses for all 180 trials of the Episodic Projection task. Each data point is the mean of 7–9 functional MRI (fMRI) participants [averaged after defining each prefrontal cortex (PFC) region within the individual] and at least 37 behavioral participants. The 2 × 2 format contrasts component processes associated with Scene Construction (*left*) and Difficulty (*right*) between 2 nearby regions: the PFC region linked to Default Network-A (DN-A) (*top*) and the PFC region linked to Frontoparietal Network-A (FPN-A) (*bottom*). A clear and robust double dissociation is observed. The PFC region falling within dorsolateral PFC (DLPFC) and linked to DN-A shows a strong positive association with Scene Construction and a negative relation to Difficulty. The large, extended lateral PFC region linked to FPN-A shows a strong positive association with Difficulty and no effect of Scene Construction. Pearson’s correlation values are displayed at *bottom left*.

Activity within the DLPFC region linked to DN-A showed a strong, positive relation to Scene Construction scores (*r* = 0.57; *P* < 0.001) and a negative relation to Difficulty scores (*r* = −0.40; *P* < 0.001). By contrast, activity within the FPN-A PFC region strongly correlated to trial Difficulty (*r* = 0.72; *P* < 0.001) and not to Scene Construction (*r* = −0.02; *P* = 0.77). Region-specific models showed that behavioral scores predicted activity in PFC regions of both DN-A [*F*(3,176) = 46.06, *P* < 0.001] and FPN-A [*F*(3,176) = 64.13, *P* < 0.001], with Scene Construction as the strongest (and only positive) predictor of activity within the DN-A region ($R_{\text{SceneConstruction}}^{2}$ = 0.30, *P* < 0.001; $R_{\text{Difficulty}}^{2}$ = 0.13, *P* < 0.001; $R_{\text{Others-Relevant}}^{2}$ = 0.01, *P* = 0.49) and Difficulty as the only significant predictor of activity within the FPN-A region ($R_{\text{Difficulty}}^{2}$ = 0.51, *P* < 0.001; $R_{\text{SceneConstruction}}^{2}$ = 0.00, *P* = 0.30, $R_{\text{Others-Relevant}}^{2}$ = 0.01, *P* = 0.94). The scatterplots of the individual trial responses in Fig. 3 illustrate these patterns. Note again that these regions of PFC were defined before examining the task data. The strong and clear relations between component processes derived from the behavioral measures and the fMRI responses are unbiased estimates.

A further post hoc exploration was conducted on the trial-level responses: the relation of Scene Construction scores and DN-A PFC regional activity was explored when restricted to only the trials in the control conditions of the original Episodic Projection task design (that is, the 90 trials from the 3 reference conditions of Ref. 43, Present Self, Past Non-Self, and Future Non-Self, designed to minimize demands on remembering). Response in the PFC region of DN-A significantly correlated with Scene Construction (*r* = 0.37; *P* < 0.001) and in the PFC region of FPN-A with Difficulty (*r* = 0.69, *P* < 0.001). Weaker relations were observed between DN-A PFC regional activity and Difficulty (*r* = 0.14, *P* = 0.18) and between FPN-A PFC regional activity and Scene Construction (*r* = 0.20, *P* = 0.05; see Supplemental Materials). Overall, a double dissociation between PFC regions associated with DN-A and FPN-A, as revealed by the original task contrasts, was also supported by trial-level data.

### A Large Region Extending from Dorsolateral Prefrontal Cortex to the Inferior Frontal Gyrus Responds to Cognitive Effort in a Domain-Flexible Manner

**Figure 4.**
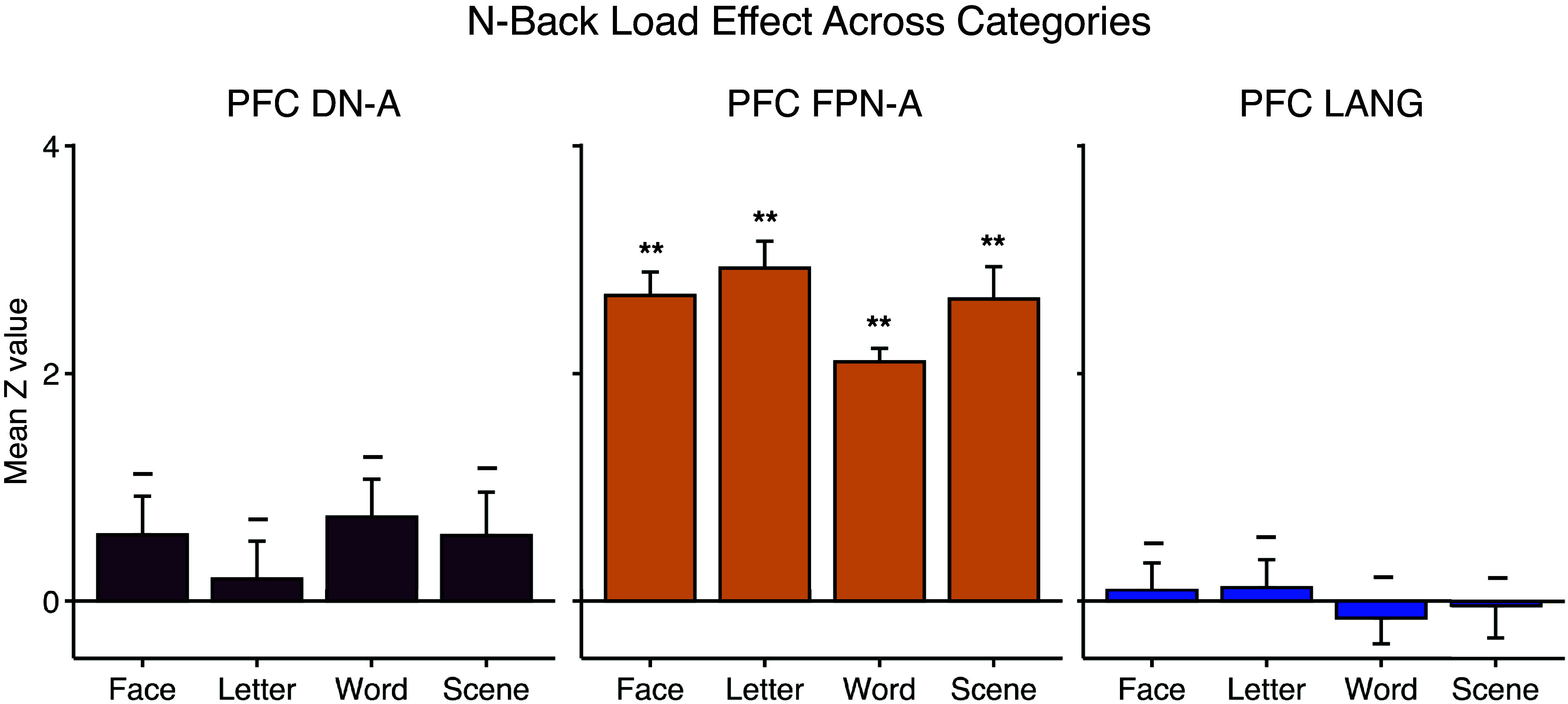
The prefrontal region associated with control responds flexibly across multiple domains. Each plot displays the mean response across the 4 stimulus conditions of the N-Back Load Effect contrast (Face, Letter, Word, and Scene) for 1 of the 3 lateral prefrontal cortex (PFC) regions: the PFC region linked to Default Network-A (DN-A) (*left*), the PFC region linked to Frontoparietal Network-A (FPN-A) (*center*), and the PFC region linked to the language network (LANG) (*right*). Note the strong response in the PFC region linked to FPN-A for each stimulus category (see also Ref. 61). The 2 other regions showed minimal response across all conditions. Error bars show SE across participants. **P < 0.001, response significantly different from 0; –*P* > 0.05.

We next probed the domain flexibility of the extended PFC region linked to FPN-A by testing whether it responded to the N-Back Load Effect contrast across additional stimulus categories (i.e., Faces, Scenes, and Words). The FPN-A region robustly increased activity to the N-Back Load Effect contrast across all stimulus categories [Letters: *t*(8) = 12.47, Faces: *t*(8) = 13.17, Scenes: *t*(8) = 9.33, Words: *t*(8) = 17.31, all *P* < 0.001; see Fig. 4]. Notably, the nearby PFC regions linked to DN-A and LANG showed a minimal N-Back Load Effect, with none of the eight contrasts significant, even with liberal statistical criteria [DN-A—Letters: *t*(8) = 0.61, Faces: *t*(8) = 1.73, Scenes: *t*(8) = 1.52, Words: *t*(8) = 2.24, all *P* > 0.05; LANG—Letters: *t*(8) = 0.49, Faces: *t*(8) = 0.40, Scenes: *t*(8) = −0.13, Words: *t*(8) = −0.65, all *P* > 0.05; Fig. 4]. The overall N-Back Load Effect contrast (averaged across all categories) showed clear and robust preferential recruitment of the PFC region linked to FPN-A over those of both DN-A [*t*(8) = 7.74, *P* < 0.001] and LANG [*t*(8) = 13.31, *P* < 0.001].

### Domain-Specialized Prefrontal Regions Spatially Juxtapose Domain-Flexible Control Regions

**Figure 5.**
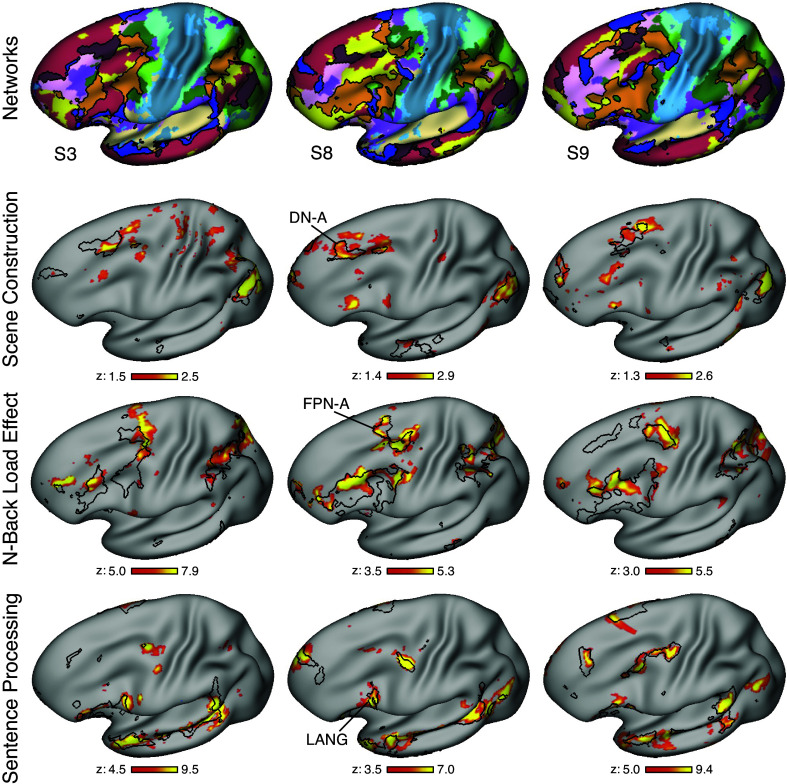
Scene Construction, N-Back Load Effect, and Sentence Processing task contrast maps align with Default Network-A (DN-A), Frontoparietal Network-A (FPN-A), and language network (LANG) regions within and beyond prefrontal cortex (PFC). Within-individual task contrast maps are displayed in relation to network boundaries in the left hemisphere. The surface view is tilted to more fully appreciate dorsolateral PFC (DLPFC). *Top*: full cerebral network estimates are shown with networks DN-A (maroon), FPN-A (orange), and LANG (blue) highlighted. These 3 networks are each individually traced as outlines in the panels below. *Second row*: task activation maps for the Scene Construction contrast are displayed in relation to the border of DN-A. *Third row*: task activation maps for the N-Back Load Effect contrast are displayed in relation to the border of FPN-A. *Bottom*: task activation maps for the Sentence Processing contrast are displayed in relation to the border of LANG. *z*-Statistical values are plotted, with thresholds varying by individual but similar within task contrasts. Note that the task activation spatial patterns vary between individuals but tend to fall within the bounds of the estimated networks, including regions in lateral PFC and also across the cortex. Maps from all individuals (including medial and lateral views in both hemispheres) are in the Supplemental Materials.

A final analysis explored functional differentiation across PFC without making assumptions about the spatial extents of the responses. We visualized the Scene Construction, N-Back Load Effect, and Sentence Processing contrast maps within each individual in relation to their a priori individually defined DN-A, FPN-A, and LANG network boundaries (Fig. 5; see also Supplemental Materials). The results revealed that the network estimates predicted well the topographic organization of the juxtaposed PFC regions, as well as numerous idiosyncratic features throughout the cortical mantle.

Specifically, the Scene Construction maps displayed alignment with DN-A in DLPFC and minimal PFC activity outside of DN-A bounds, even at relatively low thresholds. Of interest, the Scene Construction response involved a relatively circumscribed region of DLPFC that was rostral and dorsal to the major extended response of the N-Back Load Effect. Regions of FPN-A revealed overlap with the N-Back Load Effect map, and PFC LANG regions aligned almost exclusively with the Sentence Processing contrast.

Considering the contrast maps in relation to the full network estimates (Fig. 5, *top*) illustrates the specificity of the described relations and some further details. Specifically, beyond PFC, the contrast maps show network-level alignment, with regions in parietal and temporal association cortex showing similar network differentiation. The N-Back Load Effect map shows the least specific pattern, with activity extending beyond FPN-A in multiple zones (e.g., see *S3* in Fig. 5). Post hoc analysis across all 15 networks estimated by the MS-HBM revealed that, on average, when the N-Back contrast recruited a network along with FPN-A, it was most strongly a dorsal attention network (see Supplemental Materials).^5^

Overall, the patterns clearly show that PFC regions, both inferior and dorsal, can be identified that are domain-specialized components of distributed networks, dissociable from nearby control regions that occupy large portions of PFC.

## DISCUSSION

We provide evidence for multiple domain-specialized PFC regions that are distinct from (but juxtaposed with) domain-flexible regions supporting cognitive control. The domain-selective PFC regions were identified within individuals based on their participation in distinct parallel association networks. One region, located in inferior PFC, was previously highlighted by Fedorenko and colleagues (37, 51) and responded when meaningful sentences were processed. This region appears domain specialized for processing language. A second region, falling within DLPFC, is a component of a network coupled to the hippocampal formation, referred to as DN-A, that responded when mental scenes were constructed in the service of remembering and imagining the future. The domain-selective region of DLPFC did not increase its response to trial difficulty and could be dissociated from a large, juxtaposed region that behaved as predicted by models of cognitive control (increasing response to working memory load and trial difficulty and doing so across multiple stimulus domains). We discuss the implications of these findings, including a hypothesized relation to monkey anatomical data, which also support that a specific region of DLPFC may be a component of a distributed network anatomically connected with the MTL.

### Side-by-Side Regions in Prefrontal Cortex Respond Differentially to Domain-Specific Processing and Domain-Flexible Cognitive Control

A recurring debate about PFC organization revolves around whether regions participate in domain-flexible versus domain-specialized processing. Neurophysiological recordings and anatomical tracing studies in monkeys have provided support for both hypotheses (4, 21, 26, 27). Neuropsychological and neuroimaging results from humans have also yielded support for both specific and more global PFC roles (e.g., Refs. 1, 3, 29; see also Refs. 33–36, 63). The present findings, along with critical work by others (e.g., Refs. 37, 38), offer a perspective that may reconcile divergent views: certain regions within PFC may be domain specialized, distinct from adjacent domain-flexible regions.

Evidence for this perspective previously came from work on linguistic processes. Fedorenko and colleagues (37), using within-individual precision neuroimaging, demonstrated that inferior PFC regions increasing activity to meaning-based sentence comprehension are next to, but distinct from, regions behaving as domain-flexible resources. Anatomically, both sets of regions fell along the inferior frontal gyrus, at or near Brodmann areas 44/45. We replicate and extend these results, finding similar distinctions between inferior PFC regions and further demonstrating that these regions share properties with the broader networks to which they are coupled. The domain-flexible control regions are likely subregions of the larger multiple-domain network (7) (or FPN-A), which is anatomically distinct from the LANG network.

We also provide a second critical line of evidence for juxtaposed domain-specialized and domain-flexible regions in PFC, focusing on a specific region of DLPFC specialized for spatial/scene construction processes. We demonstrate that this specific region within DLPFC, coupled to the MTL, responds to remembering and imagining the future (Fig. 2). Furthermore, the component process that drives this response tracks imagining spatial content in mental scenes (Fig. 3; see Supplemental Materials). Certainly, there is more to be done to better understand the computations and boundaries of the task conditions that will elicit such responses, but all results suggest that the response in this region of DLPFC is restricted to a domain of information that is distinct from other domains.

These findings are particularly relevant to historical debate because the domain-specialized region of DN-A falls near Brodmann areas 9/46, often the focus of cognitive control investigations in humans (9, 64, 65). In monkeys, area 46 within the DLPFC (near the mid-principal sulcus) has also been the center of debate in neurophysiological studies (e.g., contrast Ref. 66 with Ref. 22; see Refs. 67 and 10 for more recent discussion).

In the human literature, convergent evidence that specific DLPFC regions might respond to domain-selective information can be found. For example, Deen and Freiwald (44) showed that DLPFC responds to mentally navigating familiar places. In our own work, if one examines the maps constructed to isolate remembering, DLPFC is often present (e.g., Refs. 43, 52). In exploring this region in detail, our present results reveal that a scene-selective region of DLPFC can be robustly dissociated from juxtaposed regions involved in domain-flexible cognitive control.

These findings thus add another example of domain selectivity in PFC. The language-specialized regions near Broca’s area are not an exception within an otherwise domain-flexible PFC. Rather, they are one example of what may be a more general principle: multiple domain-specialized processing regions exist within PFC that are distinct from the domain-flexible control regions that have been the recent emphasis of the field. These regional functional distinctions appear to reflect differences between distributed association networks. In the next section, we discuss possible anatomical origins of the selectivity of the DLPFC region involved in spatial/scene processing.

### Origins and Implications of Selectivity within Dorsolateral Prefrontal Cortex

The observation of a domain-selective region in human DLPFC involved in processing spatial/scene information during remembering and imagining is consistent with the hypothesis that specific subregions of PFC may be components of the extended hippocampal-cortical network. Several direct anatomical observations in the monkey support the possibility of such a network.

**Figure 6.**
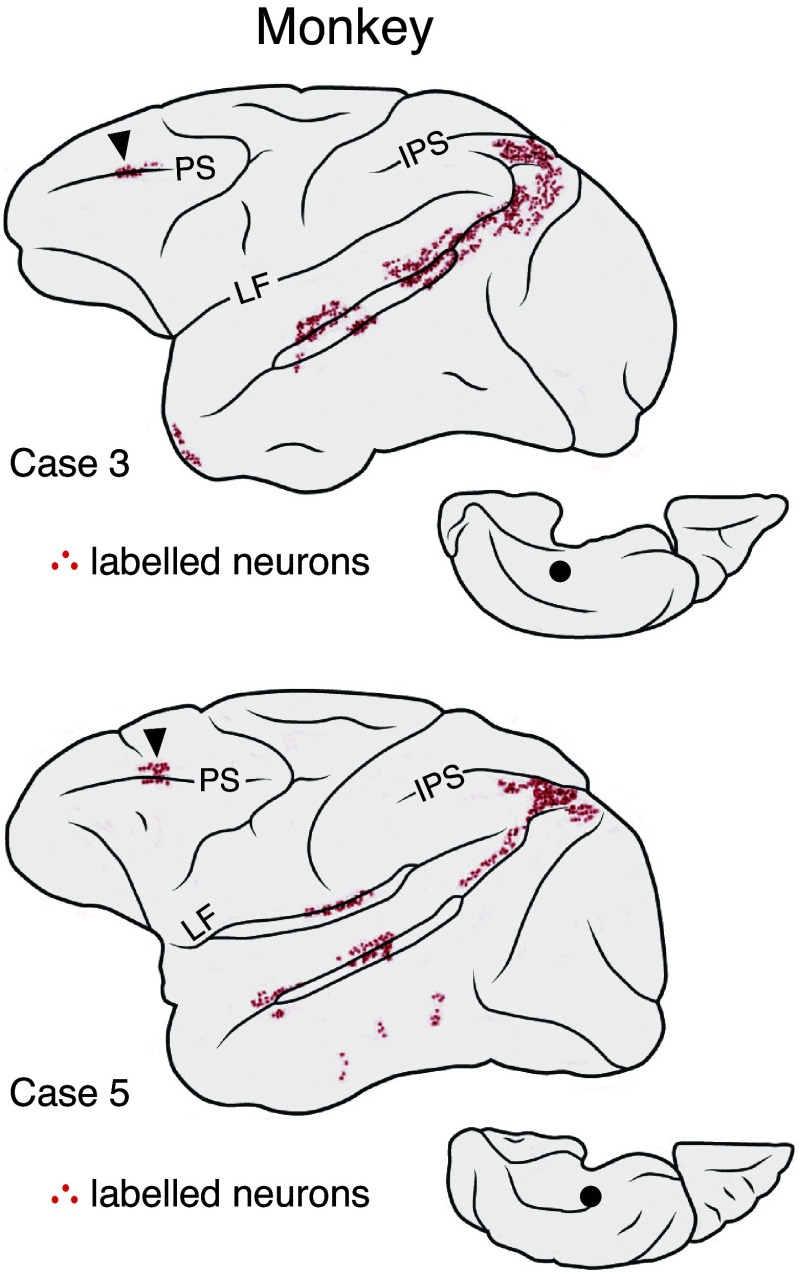
Anatomical projections in the monkey provide a candidate substrate for domain specialization in dorsolateral prefrontal cortex (DLPFC). Examples of rhesus monkey retrograde tracer injections reveal a pathway between parahippocampal and prefrontal regions. Two example cases of retrograde tracer injections placed in posterior parahippocampal gyrus reveal distributed patterns of connectivity that include a small region along mid-principal sulcus at or near area 46 (marked by arrowheads). Red dots indicate labeled neurons. *Case 3* (*top*) involves an injection between areas THO and TLO; *case 5* (*bottom*) involves an injection of area TF. Injection sites are illustrated in the ventral view *inset* at *bottom right* for each case. Reproduced from Blatt et al. (69), with permission. One possibility is that a distributed network involved in navigation and spatially relevant processing, with connectivity between the hippocampal formation and DLPFC, was shared in a distant primate ancestor and supports mental scene construction in humans, including during remembering. IPS, intraparietal sulcus; LF, lateral (Sylvian) fissure; PS, principal sulcus.

Petrides and Pandya (68) replicated, across multiple rhesus monkey cases, that injections at or near area 9/46d in PFC label parahippocampal gyrus. Similarly, in the anatomical studies of Blatt et al. (69), they describe multiple rhesus monkey cases that demonstrated projections from DLPFC to the MTL. In the cases redrawn in Fig. 6 (69), for example, retrograde label was injected into the posterior parahippocampal gyrus, with neurons labeled at and around the mid-principal sulcus near area 46. Saleem and colleagues (17) also injected retrograde label at or near area 9 in cynomolgus macaque monkeys, revealing labeled neurons in parahippocampal cortex. In a series of cases in rhesus and Japanese macaque monkeys using rabies virus (which travel polysynaptically), Hirata and colleagues (70) further injected area 46 in DLPFC and detected label in multiple portions of the MTL, including parahippocampal gyrus and entorhinal cortex. Although additional work is needed to test homologies (e.g., see Refs. 71, 72 for recent evidence that human homologs of the mid-principal sulcus are more ventral to DLPFC regions of DN-A), evidence for connectivity between the MTL and lateral PFC is consistently observed and bidirectional. Shared connectivity to other candidate regions of the hippocampal-cortical network provides further support. Pandya and colleagues (73), for example, used autoradiographic labeling of injections to the posterior cingulate gyrus (area 23) of rhesus monkeys and found distributed connectivity, including to both DLPFC and parahippocampal cortex.

As additional evidence from a New World monkey, Liu et al. (74) explored homologs of the human default network in the common marmoset monkey and emphasized that DLPFC may be a critical component. Two cases are particularly revealing (viewable at www.marmosetbrain.org). Injection of A46D (case CJ801) labels parahippocampal cortex area TH and the multiple distributed regions that parallel human DN-A, including a parietal association region near Opt, temporal association cortex, and strong label along the posterior midline including area PGM. Injection of PGM (case CJ84) labels area TH, a region near Opt, and DLPFC including prominently A46D.

Collectively, these diverse cases that span monkey species, distributed injection sites, and multiple tracing techniques all converge to suggest that there is an anatomically connected network that links the MTL with DLPFC (see Refs. 75, 76 for further description). We propose that a DLPFC region may thus derive domain-selective response properties from participation in a network involving the hippocampal formation that contributes to representation of scenes (59, 77).

The proposal of a DLPFC region as a component of a hippocampal-cortical network may also explain anatomical details of neurodegeneration in Alzheimer’s disease. Although the general pattern of atrophy in early Alzheimer’s disease has not emphasized lateral PFC involvement, a close examination reveals a specific region of DLPFC affected early in the course of disease progression. In aggregated multicenter analyses of metabolism reduction in Alzheimer’s disease, Herholz et al. (78) noted hypometabolism in a subregion of DLPFC. Cross-sectional (79, 80) and longitudinal (81, 82) atrophy estimates also note reduction in this region of DLPFC. In a telling visualization, we previously compared functional MRI memory effects with the patterns observed in estimates of Alzheimer’s disease atrophy and hypometabolism (see Fig. 6 in Ref. 81). All estimates converge to suggest that a region in DLPFC is involved, consistent with the possibility that neurodegeneration is affecting the full distributed hippocampal-cortical network that includes the region of DLPFC described in this article.

### Conclusions

Side-by-side regions of PFC, participating in distinct parallel association networks, differentially support domain-flexible cognitive control and domain-specialized processing. One domain-specialized region, within DLPFC, is a component of a distributed network that involves the hippocampal formation and is active during remembering and imagining the future. Analysis of trial-level variation further suggests that the region is active when imagining mental scenes, consistent with specialization of the network for processing space and the use of such spatial representation during acts of remembering.

## DATA AVAILABILITY

Parcellations and task maps from the figures are available on Balsa (https://balsa.wustl.edu/study/n8PDP). Normalized trial-level strategy ratings associated with the Episodic Projection task (also from Exp. 2 in Ref. 52) are posted on Harvard Dataverse (https://doi.org/10.7910/DVN/NZKRNS).

## SUPPLEMENTAL MATERIAL

10.6084/m9.figshare.24262972 Supplemental Materials: https://doi.org/10.6084/m9.figshare.24262972.

## GRANTS

This work was supported by Kent and Liz Dauten, NIH Grants MH124004 and P50MH106435, and Shared Instrumentation Grant S10OD020039. For L.M.D., this work was supported by the National Science Foundation GRFP under grant DGE1845303, The Pershing Square Fund for Research on the Foundations of Human Behavior, and the Sigma Xi Society (GIAR G20201001117410844). This material is based upon work supported by the National Science Foundation under grant DRL2024462. For W.S., this work was supported by the Paul and Daisy Soros Foundation.

## DISCLOSURES

No conflicts of interest, financial or otherwise, are declared by the authors.

## AUTHOR CONTRIBUTIONS

L.M.D. and R.L.B. conceived and designed research; L.M.D. and W.S. performed experiments; L.M.D., W.S., and R.L.B. analyzed data; L.M.D., W.S., and R.L.B. interpreted results of experiments; L.M.D. and R.L.B. prepared figures; L.M.D. and R.L.B. drafted manuscript; L.M.D., W.S., and R.L.B. edited and revised manuscript; L.M.D., W.S., and R.L.B. approved final version of manuscript.

## Footnotes

1 The network here labeled Frontoparietal Network-A (FPN-A) has been variably named in the literature, leading to confusion (see Ref. 57 vs. Ref. 58). Here we adopt the MS-HBM labeling convention used by Xue et al. (58). The network here termed FPN-A is likely the same as that identified as FPN-B by Braga et al. (42, 57) and DiNicola et al. (52).

2 The MS-HBM works robustly in our experience when run in small groups that help to initialize the parameters. For any given individual, testing in our lab (61) showed that results are nearly identical when the person is included in one group versus another. As a result, we run the network estimation procedure in groups of five or six participants and then utilize their within-individual a priori network estimates in a prospective fashion for all further analyses (see also Ref. 61).

3 As described previously, Difficulty showed a strong correlation to scores on two additional composites, termed Self-Relevant and Autobiographical, introducing the potential for multicollinearity (52). These composites were removed from interpreted models in the prior work and here.

4 Note that the tests are one -tailed because of the clear direction of the hypothesis. The *t* tests are not corrected for multiple comparisons because they explore distinct convergent predictions of the hypothesis and thus reinforce one another, rather than providing independent opportunities to support the hypothesis. Nonetheless, it is worth noting the effects would be significant even if a two-tailed test were warranted and the most conservative multiple comparison correction applied (e.g., Bonferroni correction).

5 These results also highlight the intriguing and open question of what role the juxtaposed network, FPN-B, might play in control-relevant or yet-unexplored processes.
